# Isolation of the Secondary Building Unit of a 3D Metal–Organic Framework through Clip-Off Chemistry, and Its Reuse To Synthesize New Frameworks by Dynamic Covalent Chemistry

**DOI:** 10.1021/jacs.4c09077

**Published:** 2024-09-30

**Authors:** Dongsik Nam, Jorge Albalad, Roberto Sánchez-Naya, Sara Ruiz-Relaño, Alba Cortés-Martínez, Yunhui Yang, Judith Juanhuix, Inhar Imaz, Daniel Maspoch

**Affiliations:** † Catalan Institute of Nanoscience and Nanotechnology (ICN2), CSIC and The Barcelona Institute of Science and Technology, Campus UAB, Bellaterra, 08193 Barcelona, Spain; ‡ Department of Chemistry, Autonomous University of Barcelona (UAB), Campus UAB, Bellaterra, 08193 Barcelona, Spain; § Alba Synchrotron Light Facility, Cerdanyola del Vallès, 08290 Barcelona, Spain; ∥ ICREA, Passeig Lluís Companys 23, 08010 Barcelona, Spain

## Abstract

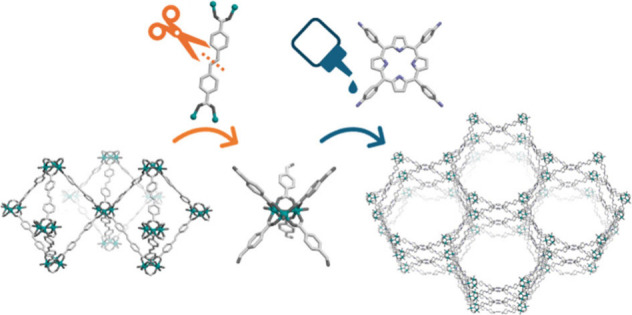

Herein, we present a novel methodology for synthesizing metal clusters or secondary building units (SBUs) that are subsequently employed to construct innovative metal–organic frameworks (MOFs) via dynamic covalent chemistry. Our approach entails extraction of SBUs from preformed MOFs through complete disassembly by clip-off chemistry. The initial MOF precursor is designed to incorporate the desired SBU, connected exclusively by cleavable linkers (in this study, with olefinic bonds). Cleavage of all the organic linkers (in this study, via ozonolysis under reductive conditions) liberates the SBUs functionalized with aldehyde groups. Once synthesized, these SBUs can be further reacted with amines in dynamic covalent chemistry to build new, rationally designed MOFs.

Metal–organic frameworks (MOFs) are crystalline porous materials composed of metal ions or clusters connected by organic linkers. The design approach for MOFs has gradually shifted from serendipity to rational methods.^1,2^ Among synthetic strategies, the use of secondary building units (SBUs) has been pivotal in the systematic design and construction of MOFs.^1^ These SBUs, which are formed by coordinating metal ions or clusters with organic linkers, determine the overall topology and properties of the resulting MOF, including its porosity and chemical reactivity. Today, myriad SBUs with diverse compositions, geometries, sizes and connectivities are well-documented.^3^ Thus, predicting their formation by combination of the basic metal and organic building blocks is essential for the rational design of MOFs. However, chemical reactions involving metal ions remain complex and sensitive to experimental conditions,^4,5^ often necessitating synthetic optimization for target SBUs, which relies heavily on trial and error.

One strategy to reduce uncertainty in this chemistry involves the use of presynthesized SBUs for constructing MOFs.^6−11^ Early efforts included synthesizing discrete Zr_6_-based SBUs and subsequently forming extended Zr-based MOFs through linker exchange. For example, Guillerm et al. synthesized a Zr_6_-based cluster with monocarboxylate ligands and assembled UiO-66-type MOFs by introducing additional dicarboxylate linkers.^7^ Subsequently, Zr_12_ and Zr_16_ clusters were also utilized to create Zr-based MOFs via linker exchange, as demonstrated by Bezrukov et al. and Hou et al.^9,10^ However, in these studies, while the Zr clusters were incorporated into the resulting MOFs, the linker exchange processes could alter the coordination environment and final connectivity of the clusters, thereby introducing uncertainty into predicting formation of the resultant MOFs.

Different approaches involving preformed SBUs have been demonstrated using dynamic covalent chemistry, commonly employed in the synthesis of covalent-organic frameworks (COFs).^12−22^ Nguyen et al. synthesized Ti-based MOFs composed of Ti_6_ SBUs linked via imine condensation.^23,24^ Although these MOFs were formed through a one-pot reaction, this study showed promise by combining the chemistry of MOFs and COFs. Alternatively, stepwise synthesis using preformed SBUs was explored.^12−18^ In 2019, Xu et al. synthesized a MOF by connecting ditopic amino-functionalized polyoxometalate with 4-connected aldehyde-based linkers.^12^ In 2020, Wei et al. and Li et al. utilized Cu(I)-based trimeric SBUs featuring three terminal aldehyde groups to connect with ditopic amine linkers.^16,17^ Despite these recent advances, the use of preformed SBUs to synthesize extended MOFs remains in its early stages, limited to only a few types of metal clusters. This limitation largely stems from the challenge of synthesizing metal clusters with the available functional linkers required for their use as extendable SBUs in constructing the desired MOFs.

Herein, we report a new approach for synthesizing metal clusters or SBUs, which involves extracting them from preformed MOFs through complete disassembly via clip-off chemistry (Figure 1a).^25−27^ To achieve this, the initial MOF precursor must incorporate the targeted SBU, connected exclusively via linkers containing cleavable bonds (in this study, olefinic bonds) (Figure 1b). Cleavage of all organic linkers via reductive ozonolysis releases the SBUs functionalized with terminal aldehyde groups. Thanks to these functional groups, these presynthesized SBUs can then undergo dynamic imine condensation to form novel MOFs with predictable topologies.

**Figure 1:**
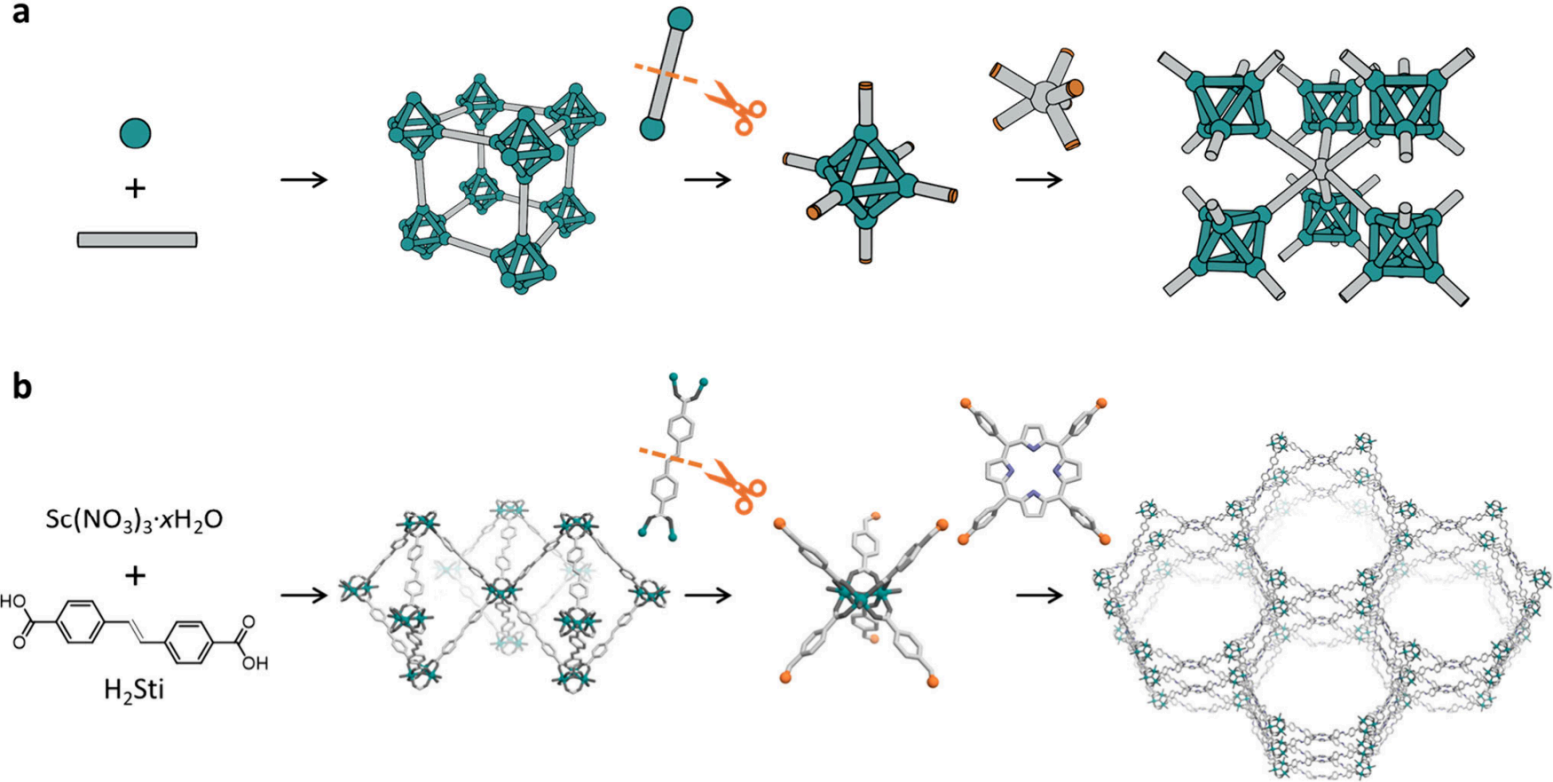
(a) Schematic illustrating our synthetic approach that entails isolating a SBU from a 3D MOF using clip-off chemistry. This SBU can then be reused to construct an extended framework. (b) Schematic illustrating application of our strategy to synthesize a 6-connected trimeric Sc^3+^ cluster terminated with aldehyde groups, and its subsequent extension through a 4-connected amine linker to form a 3D **stp** MOF.

As a proof-of-concept to demonstrate our approach, we targeted the clip-off synthesis of the typical trimeric metal cluster with the general formula M_3_(μ_3_-Ο)(−COO)_6_ (M = Fe^3+^, Sc^3+^, Al^3+^, In^3+^, Cr^3+^, V^3+^, Mg^2+^, Co^2+/3+^, Ni^2+^, Mn^2+/3+^, Ga^3+^), which has been among the most explored and versatile SBUs since this field began.^28−38^ This SBU adopts a triangular prismatic geometry, establishing six connections to organic linkers. Among all metal ions, we selected the trimeric cluster, composed of Sc^3+^, given the absence of similar clusters with available functional groups. In fact, to date, only one example using amino acids has been reported in the Cambridge Structural Database (No. CCDC-103179).^39^

When these 6-connected trimeric clusters are connected via six ditopic linkers, various archetypal MOFs and topologies can be formed, such as MIL-101 (**mtn** topology) or MIL-88 (**acs** topology).^6,34^ Among these, we chose to synthesize a MOF precursor isoreticular to MIL-88, as it is known that the latter can be assembled using ditopic linkers of varying lengths.^37^ One of these isoreticular MIL-88 analogs comprising long ditopic linkers is MIL-126, which contains two interpenetrated **acs** nets built up by connecting the Sc^3+^ clusters through biphenyl-4,4′-dicarboxylate linkers (Figure S1).^40−42^ Accordingly, we attempted to synthesize a MIL-126 analog using the targeted Sc^3+^ clusters through a ditopic cleavable linker of similar length, 4,4′-stilbenedicarboxylic acid (H_2_Sti). The reaction of scandium nitrate hydrate and H_2_Sti in *N*,*N*-dimethylformamide (DMF) and concentrated HCl afforded a white crystalline material that was characterized to confirm its isoreticular relationship with MIL-126. To verify this, we used Materials Studio^43^ to construct a model framework based on the structure of the two-interpenetrated MIL-126 with the *P*4_3_2_1_2 space group.^40^ Geometric energy minimization of the model was performed with the Forcite module. The simulated powder X-ray diffraction (PXRD) pattern of the model closely matched the experimental pattern of the MIL-126 analog (Figure S2). A full-profile Pawley fitting was conducted, yielding final unit-cell parameters of *a* = 24.993(2) Å and *c* = 42.414(6) Å, with good agreement factors (*R*_p_ = 1.4% and *R*_wp_ = 3.5%), confirming the formation of the anticipated framework.

Once we had synthesized our MOF precursor, we then explored using clip-off chemistry to fully disconnect the MIL-88/MIL-126 type framework. We reasoned that this would enable synthesis of the trimeric Sc^3+^ oxocluster with molecular formula [Sc_3_O(COOC_6_H_4_CHO)_6_(H_2_O)_2_(OH)], formed by six 4-formylbenzoate linkers. To this end, our MOF precursor was dispersed in methanol and exposed to a constant ozone flux (20 g Nm^−3^) for 5 min at −10 °C (Figure 2a). The suspension was stirred for further 2 min with dimethyl sulfide (DMS) as the reducing agent.^44^ The mixture was stirred for another hour at room temperature. Next, a clear supernatant was filtered from the suspension, and then concentrated in vacuo to afford a white solid (yield = 80%). Proton nuclear magnetic resonance (^1^H NMR) spectrum of the solid showed a peak at 10.04 ppm, characteristic of aldehyde groups, and broad peaks at 8.54–7.65 ppm, attributed to the aromatic signals of 4-formylbenzoate linkers, resulting from the cleavage of Sti (Figures 2a and 2b). Moreover, we attributed this peak broadening to the presence of metal–organic Sc^3+^ complexes, as typically observed in metal–organic cages.^45^ To further confirm the presence of 4-formylbenzoate in the solid, we digested it with a cesium fluoride solution in DMSO-*d*_6_/D_2_O, from which the ^1^H NMR spectrum unambiguously confirmed the exclusive presence of free 4-formylbenzoic acid (Figure 2c). Finally, the formation and isolation of the expected Sc^3+^ cluster was corroborated by matrix-assisted laser desorption/ionization mass spectrometry (MALDI-MS). The spectrum exhibited two main peaks, at *m*/*z* = 1045.0 and 1123.0, corresponding to the theoretical values (*m*/*z* = 1045.0 and 1123.0) of the expected Sc^3+^ cluster with the molecular formula [Sc_3_O(COOC_6_H_4_CHO)_6_]^+^ and [Sc_3_O(COOC_6_H_4_CHO)_6_]^+^·DMSO (Figures 2d and S3).

**Figure 2:**
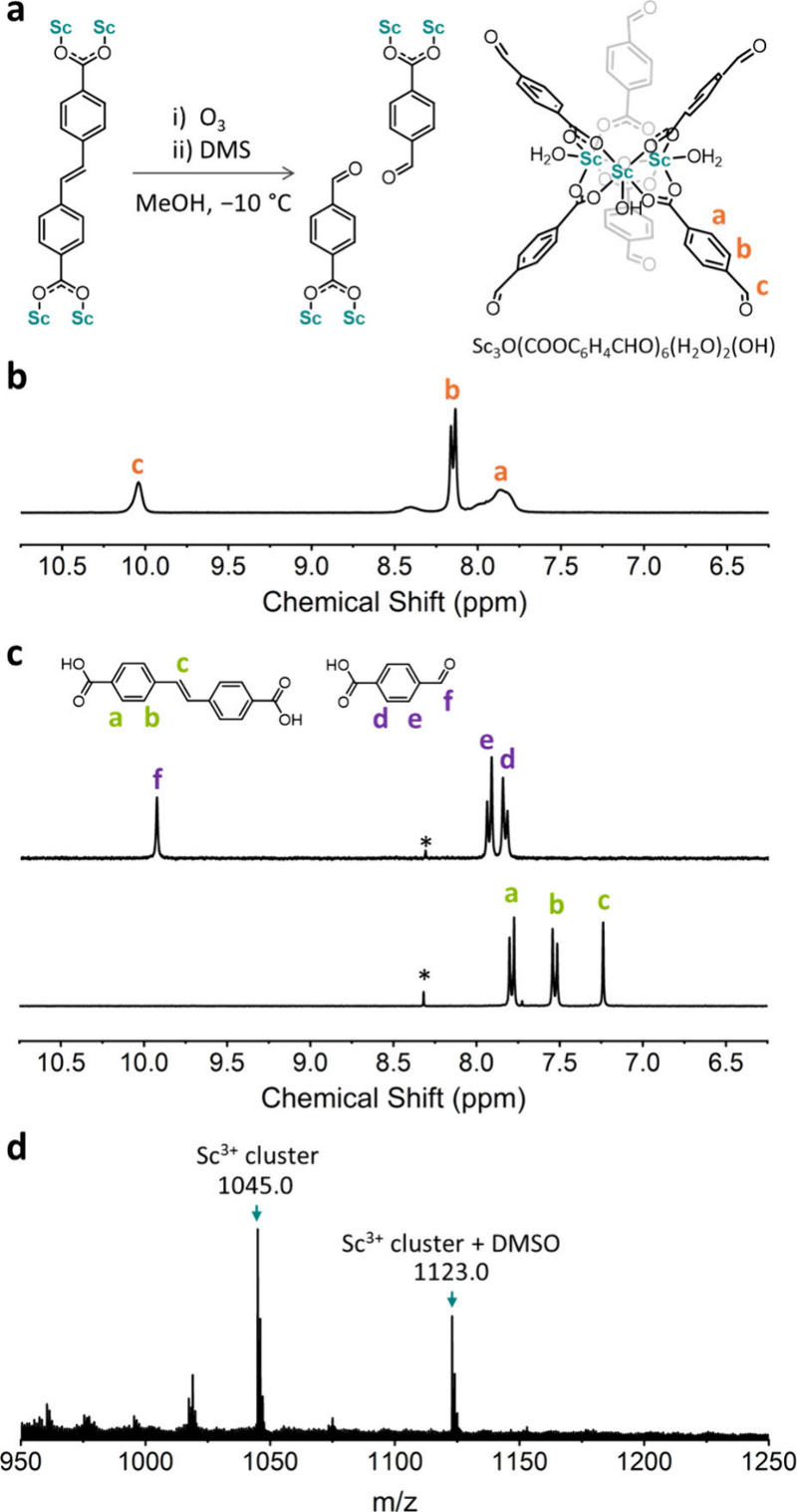
(a) Cleavage of the Sti linker under reductive conditions (left), and illustration of the Sc^3+^ cluster with terminal aldehyde groups (right). (b) ^1^H NMR spectrum of the Sc^3+^ cluster in DMSO-*d*_6_. (c) ^1^H NMR spectra of the digested MOF precursor (bottom) and the Sc^3+^ cluster (top). Asterisks indicate formic acid. (d) MALDI-MS for the Sc^3+^ cluster.

We next sought to demonstrate the utility of this synthesized Sc^3+^ cluster by using it as a 6-connected building unit, given its six terminal aldehyde groups. To achieve this, we aimed to extend its functionality with amine-based linkers through imine condensation to create novel MOFs. A critical consideration in this process is the stability of the Sc^3+^ cluster during imine condensation reactions, which often require acetic acid as a catalyst for imine-bond formation.^46−48^ Indeed, exposure of the synthesized cluster to 6 M acetic acid in DMF-*d*_7_ led to its complete decomposition, resulting in peaks corresponding only to free 4-formylbenzoic acid (Figure S4). Alternatively, we chose Sc(OTf)_3_ as a Lewis acid catalyst, renowned for its ability to facilitate the formation of imine-based COFs under milder conditions.^49^ Under these modified conditions, the Sc^3+^ cluster exhibited stability, as confirmed by ^1^H NMR spectroscopy (Figure S4). Following this approach, we investigated the feasibility of imine-bond formation by combining the Sc^3+^ cluster, featuring six terminal aldehyde groups, with aniline (Figure 3a). The reaction was run with 0.02 equiv. of Sc(OTf)_3_ in DMF-*d*_7_ at 70 °C for 3 days. After 1 day, the formation of *N*-(4-carboxybenzylidene)aniline via imine bond formation was evidenced by peaks at 8.74, 8.30, 8.10, 7.45, and 7.33 ppm in the ^1^H NMR spectrum (Figure 3b). Over the next 2 days, similar ^1^H NMR spectra were obtained, showing a total conversion of 80% (Figure S5). Additionally, peak broadening in these spectra suggested no degradation of the cluster species. Imine bond formation, and stability of the Sc^3+^ cluster, during this process were each confirmed by MALDI-MS, revealing peaks corresponding to the Sc^3+^ cluster having reacted with six, five, four, three, or two aniline molecules at *m*/*z* = 1495.5, 1420.4, 1345.3, 1270.3, and 1195.4, respectively (Figure 3c and Table S1). These values closely matched the theoretical values of *m*/*z* = 1495.3, 1420.2, 1345.2, 1270.1, and 1195.1. Formation of *N*-(4-carboxybenzylidene)aniline was confirmed by ^1^H NMR analysis of a digested sample (Figure S6). Overall, these findings confirmed that our aldehyde-terminated Sc^3+^ cluster could undergo Schiff-base condensations with amines.

**Figure 3:**
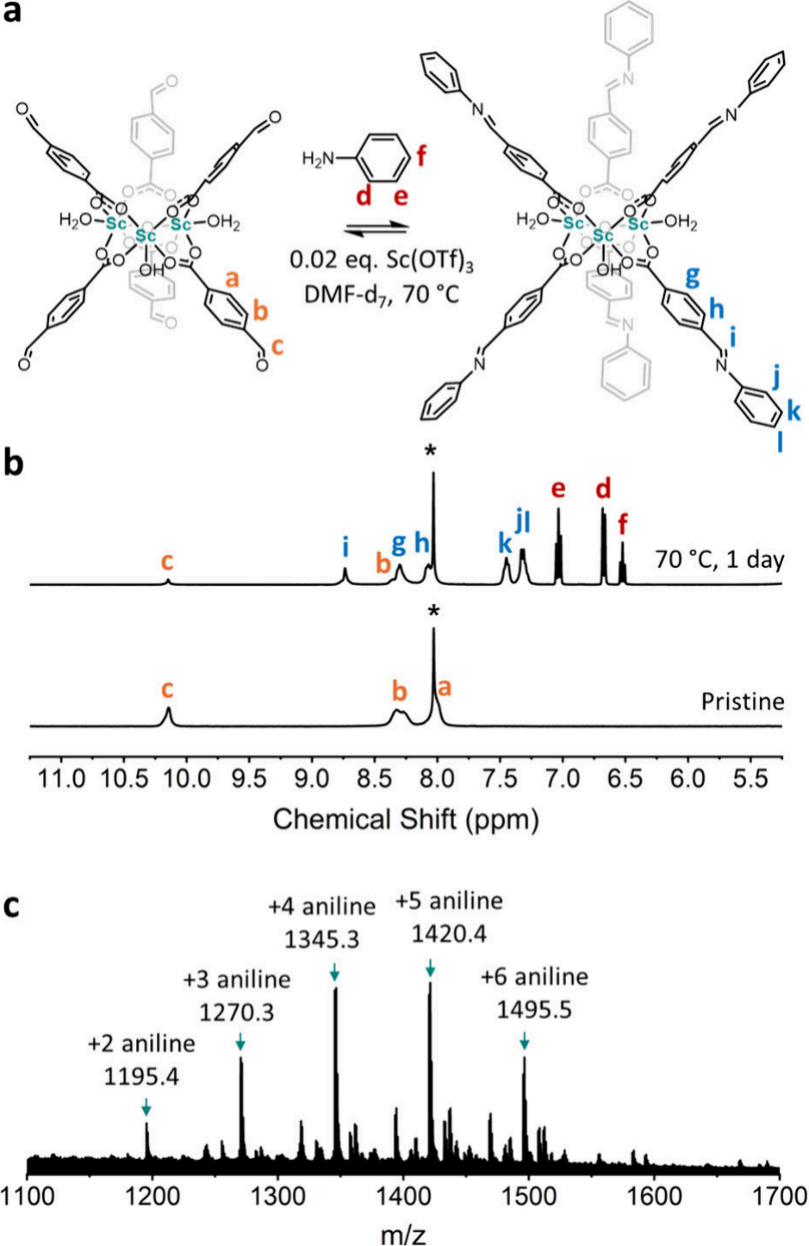
(a) Imine condensation between the Sc^3+^ cluster and aniline, using Sc(OTf)_3_. (b) ^1^H NMR spectra of pristine Sc^3+^ cluster (bottom), and the reaction (70 °C, 1 day) with aniline in DMF-*d*_7_ (top). Asterisks indicate DMF. (c) MALDI-MS after reacting the Sc^3+^ cluster, aniline and Sc(OTf)_3_.

Next, we aimed to extend the 6-connected Sc^3+^ cluster with a 4-connected amine linker such as 5,10,15,20-tetrakis(4-aminophenyl)porphyrin (TAPP), which we envisioned would lead to a new 3D MOF with large pores and an underlying **stp** topology. However, our initial attempts to reproduce the above-mentioned conditions (using 0.02 equiv. Sc(OTf)_3_, as typically employed in the synthesis of 2D-COFs) were unsuccessful, yielding only amorphous solids. At this point, we hypothesized that forming a crystalline 3D framework from highly connected aldehyde and amine building units would require greater reversibility in the imine chemistry to avoid any kinetic trapping that would lead to amorphous polymers. Interestingly, to the best of our knowledge, the synthesis of 3D COFs using Sc(OTf)_3_ and both building blocks having a connectivity higher than 2 has never been demonstrated.^50,51^ To this end, we increased the amount of Sc(OTf)_3_ to accelerate the forward reaction, and incorporated aniline as a modulator competing with TAPP. We tested different stoichiometries and conditions, eventually obtaining a highly crystalline powder by using aldehyde-terminated Sc^3+^ cluster; TAPP; 0.2 equiv. Sc(OTf)_3_; 9 equiv. aniline; dioxane/DMF (7:1 v/v) as solvent; 85 °C as the temperature; and 3 days as the time (Figure 4a). Field-emission scanning electron microscopy (FESEM), energy-dispersive X-ray (EDX) mapping, and X-ray photoelectron spectroscopy (XPS) of this solid revealed the formation of rodlike crystals (size: ∼1 μm × 4 μm) with a homogeneous distribution of scandium, oxygen, and carbon, where the Sc ions retain the same oxidation state as in the original cluster (Figures S7 and S8). XPS also confirmed the presence of porphyrin moieties in the crystals. Additionally, characterization of this solid using ^13^C cross-polarization magic angle spinning (CP-MAS) solid-state NMR confirmed the formation of imine bonds, with a characteristic imine carbon signal at ∼161 ppm (Figure S9).^52^

**Figure 4:**
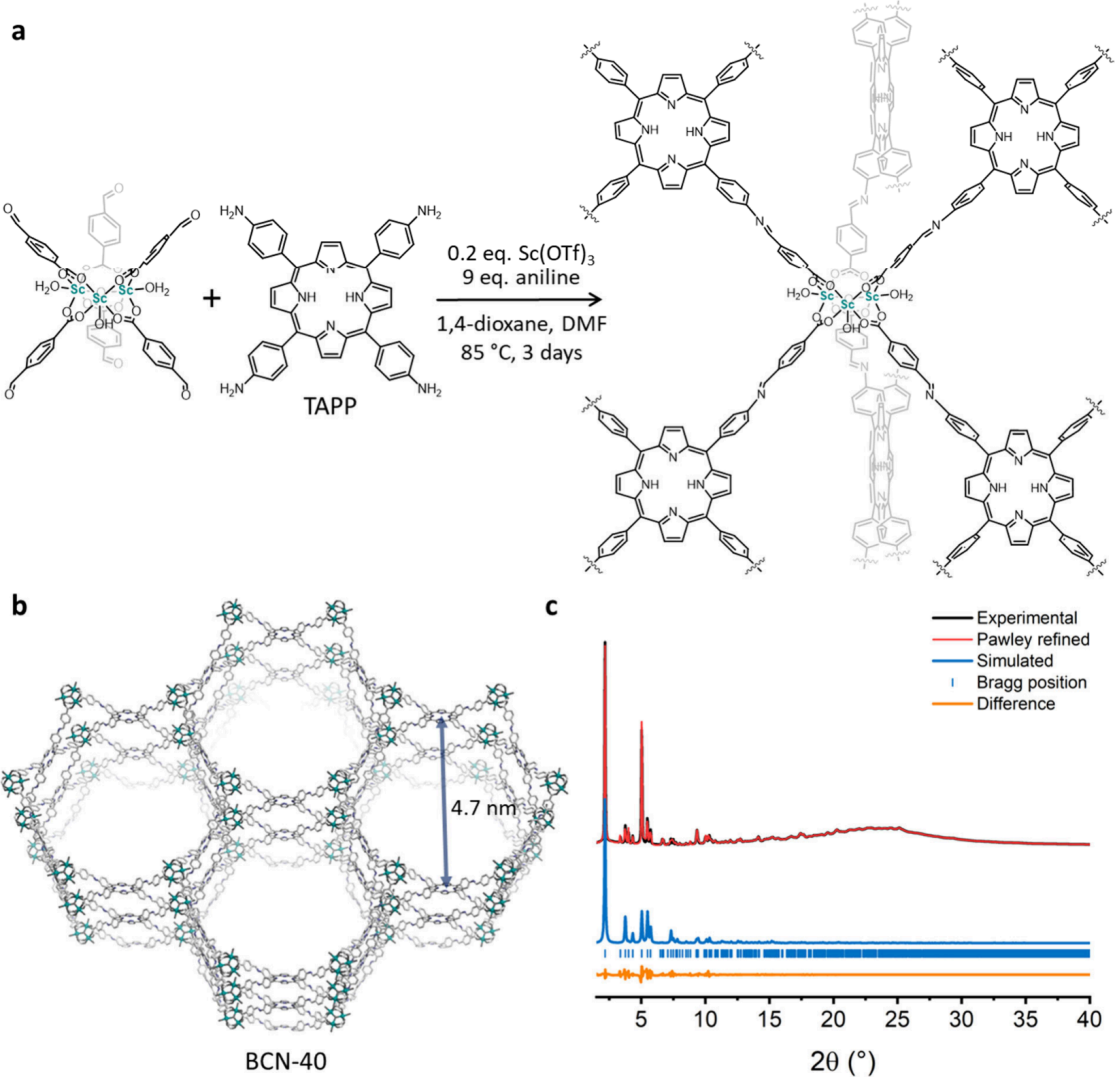
(a) Schematic for synthesis of BCN-40. (b) Proposed structure of BCN-40, featuring 4.7 nm-sized hexagonal channels. (c) PXRD pattern of BCN-40 in methanol, and the refined Pawley fitting.

To confirm the structural identity of the solid, we used Materials Studio to build and geometrically optimize a model of the target structure.^43^ The proposed structure, named BCN-40, was built up from the 6-connected Sc^3+^ clusters linked by 4-connected planar TAPP, forming 1D hexagonal channels as large as 4.7 nm (Figure 4b). Remarkably, this theoretical pore width is the largest reported for **stp** MOFs since that of 3.1 nm had been observed in PCN-600 (Table S2).^53^ A full profile Pawley fitting based on the model revealed final unit-cell parameters of *a* = 47.06(21) Å and *c* = 26.21(12) Å with good agreement factors (*R*_p_ = 1.6% and *R*_wp_ = 4.4%) (Figure 4c). The simulated PXRD pattern with these cell parameters matched well with the experimental pattern of the noninterpenetrated structure (Figure S10), confirming the successful synthesis of the expected **stp** 3D BCN-40. Porosity measurements after supercritical CO_2_ activation revealed an N_2_ uptake of 120 cm^3^ g^–1^ at 77 K (Figure S11), indicating poor porosity, a common issue in mesoporous MOFs due to their weak mechanical stability after solvent removal.^54^ However, in solution, mesopore accessibility in BCN-40 was confirmed through the adsorption of Vitamin B_12_, a model molecule (dimensions: 1.41 nm × 1.83 nm × 1.14 nm) often used to evaluate pore accessibility in mesoporous MOFs (Figure S12).^55,56^ A total uptake of 0.24 mg of Vitamin B_12_ per mg of BCN-40 was observed after 16 h of incubation.^55^

In summary, our results demonstrate that preformed SBUs or metal clusters within MOFs can be synthesized using clip-off chemistry and subsequently employed as building units for novel MOFs via dynamic covalent chemistry. Given the large variety of SBUs and clusters in MOFs, some of which only exist in those frameworks, our approach will provide access to isolated, previously inaccessible clusters and more-complex building units at the molecular level.

## Data Availability

Supporting structure modeling of MIL-126 analog, BCN-40 and interpenetrated BCN-40 are available (for comparison purposes).

## Supporting Information Available

Detailed experimental section and characterization including PXRD diffractograms, ^1^H NMR spectra, MALDI-ToF data, FESEM images, EDX elemental maps, XPS spectra, ^13^C CP-MAS solid-state NMR spectrum, N_2_ isotherm, and UV–vis spectrum (PDF)
