# Supplementary material for: Isolation of the Secondary Building Unit of a 3D Metal–Organic Framework through Clip-Off Chemistry, and Its Reuse To Synthesize New Frameworks by Dynamic Covalent Chemistry
Source: J Am Chem Soc. 2024 Sep 30;146(40):27255–61. doi: 10.1021/jacs.4c09077 (PMC11468772; doi:10.1021/jacs.4c09077)
Supplement: Supplementary file 1 — ja4c09077_si_001.pdf [file ja4c09077_si_001.pdf]

Supporting Information for

Isolation of the Secondary Building Unit of a 3D Metal-Organic Framework through Clip-off Chemistry, and its Re-Use to Synthesize New Frameworks by Dynamic Covalent Chemistry

Dongsik Nam,[1,2] Jorge Albalad,[1,2] Roberto Sánchez-Naya,[1,2] Sara Ruiz-Relaño,[1,2] Alba Cortés-Martínez,[1,2] Yunhui Yang,[1,2] Judith Juanhuix,[3] Inhar Imaz,*[1,2] and Daniel Maspoch*[1,2,4]

[1]Catalan Institute of Nanoscience and Nanotechnology (ICN2), CSIC and The Barcelona Institute of Science and Technology, Campus UAB, Bellaterra, 08193 Barcelona, Spain.

[2]Department of Chemistry, Autonomous University of Barcelona (UAB), Campus UAB, Bellaterra, 08193 Barcelona, Spain.

[3]Alba Synchrotron Light Facility, Cerdanyola del Vallès, 08290 Barcelona, Spain.

[4]ICREA, Passeig Lluís Companys 23, 08010 Barcelona, Spain.

*Corresponding authors: inhar.imaz@icn2.cat, daniel.maspoch@icn2.cat

Table of Contents

Section S1. Materials and methods ... S3
S1.1 Chemicals and reagents ... S3
S1.2 Instruments ... S3
Section S2. Experimental procedures ... S5
S2.1 Synthesis of MIL-126 analog ... S5
S2.2 Synthesis of trimeric Sc^{3+} cluster and stability test ... S5
S2.3 Extension of trimeric Sc^{3+} cluster ... S6
S2.4 Vitamin B_{12} adsorption in BCN-40 ... S6
Section S3. Supplementary figures and tables ... S8
References ... S22

Section S1. Materials and methods

S1.1 Chemicals and reagents

Scandium(III) nitrate hydrate ($Sc(NO_3)_3 \cdot xH_2O$), *N*,*N*-dimethylformamide (DMF), concentrated hydrochloric acid (HCl), acetic acid, acetone, diethyl ether and methanol (MeOH) were purchased from Thermo Fisher Scientific. 4,4'-Stilbenedicarboxylic acid (H_2Sti) was purchased from Fluorochem. Scandium triflate ($Sc(OTf)_3$), aniline, 1,4-dioxane, dimethyl sulfide (DMS), 4-formylbenzoic acid, cesium fluoride (CsF), and vitamin B_{12} were purchased from Merck. 5,10,15,20-Tetrakis(4-aminophenyl)porphyrin (TAPP) was purchased from TCI chemicals. All deuterated solvents were purchased from Eurisotop. All the chemicals were used as received without further purification. The deionized water from all the aqueous solutions in the article was obtained from a Milli-Q® system (18.2 MΩ·cm)

S1.2 Instrumentation

Synchrotron PXRD data were obtained at the BL13-XALOC beamline at the ALBA synchrotron[1] with a wavelength of 0.8765 Å for MIL-126 analog and 0.9792 Å for BCN-40. Capillaries of 0.7 mm inner diameter were prepared with MIL-126 suspension in DMF and BCN-40 suspension in MeOH. Data were collected with a PILATUS3 detector. PXRD patterns were radially integrated using FIT2D program.[2]

Proton Nuclear Magnetic Resonance (^{1}H NMR) spectra were recorded in Bruker Avance NEO 300 MHz Spectrometer and Bruker Avance NEO 400 MHz Spectrometer at "Servei de Ressonància Magnètica Nuclear" from Autonomous University of Barcelona (UAB).

Ozonolysis was conducted using a GHBZO3-E Commercial Ozone Generator from ZonoSistem equipped with ozone analyzer UVOZ-1200.

Matrix-Assisted Laser Desorption/Ionization Mass Spectrometry (MALDI MS) was performed using a 4800 Plus MALDI TOF/TOF (ABSCIEX – 2010). Trans-2-[3-(4-tert-butylphenyl)-2-methyl-2-propenylidene]malononitrile (DCTB) was used as ionization matrix.

Before mixing with the matrix, trimeric Sc^{3+} cluster was dissolved in acetone, and DMF solution of Sc cluster reacted with aniline was diluted with acetone (DMF:acetone = 1:9, v/v).

Field-Emission Scanning Electron Microscopy (FESEM) and Energy Dispersive X-ray (EDX) elemental mapping were performed using a SEM Quanta 650 FEM.

X-ray Photoelectron Spectroscopy (XPS) data was acquired using SPECS PHOIBOS 150 hemispherical energy analyser.

Solid-State ^{13}C Cross-Polarization Magic Angle Spinning (CP-MAS) NMR spectrum was collected on a Bruker Avance III 9.4T spectrometer equipped with a double channel 4.0 mm MAS probe.

N_2 Adsorption isotherm was collected at 77 K using ASAP 2460 (Micromeritics). Temperature was controlled by using a liquid nitrogen bath. Prior to the measurement, the sample was activated with supercritical CO_2.

Supercritical CO_2 Activation was performed using Laboratory Supercritical Fluid Equipment SFE15 mL (Extratex Supercritical Fluid Innovation).

Ultraviolet-visible (UV-Vis) spectra were acquired using a Thermo Scientific^{TM} NanoDrop 200 at room temperature (*ca*. 25 ºC).

Section S2. Experimental procedures

S2.1 Synthesis of MIL-126 analog

$Sc(NO_3)_3 \cdot xH_2O$ (85 mg, 0.37 mmol) and DMF (5 mL) were added to a 10 mL vial and sonicated. Concentrated HCl (30 μL) and H_2O (50 μL) were sequentially added to the solution, which was then heated in an oven at 100 °C for 18 hours. Separately, H_2Sti (75 mg, 0.28 mmol) was dispersed in DMF (5 mL) in a 20 mL vial with an aluminum-lined cap and sonicated for 3 minutes. Then, the metal solution was added into this linker suspension. After vigorously shaking the resulting mixture, slightly viscous suspension was obtained. The sample was then heated in an oven at 120 °C for 2 days. After cooling to room temperature, the resulting white crystalline powder was washed with fresh DMF (10 mL x 3) and stored in DMF prior to further use.

S2.2 Synthesis of trimeric Sc^{3+} cluster and stability test

Synthesis of Sc^{3+} cluster via ozonolysis of MIL-126 analog. One vial of MIL-126 analog stored in DMF was transferred to a centrifuge tube and washed with MeOH (10 mL x 3) using centrifugation (7000 rpm, 2 min). After shaking vigorously, 4 mL of the suspension was moved to a Pyrex tube (13 x 100 mm) equipped with a magnetic stirring bar. The tube was then sealed with a rubber septum and immersed in an acetone/ice bath (−10 °C). An ozone (O_3) gas flux at a concentration of 20 $g \cdot Nm^{-3}$ was bubbled through a syringe into the suspension for 5 min. Then, the voltage was set to 0, so that O_2 gas was bubbled for 2 min in order to remove excess O_3. After stopping the gas flow, DMS (200 μL) was injected into the suspension, and the mixture was then stirred for 2 min at −10 °C. The tube was moved to room temperature and stirred for 1 h. A clear supernatant was collected by filtering residual solids through a syringe filter (0.45 μm pore size). (Note: It is not recommended to store the supernatant overnight because insoluble precipitates might appear). The supernatant was moved to a 10 mL vial, and slowly evaporated using a rotary evaporator at 35 °C. Thin colorless transparent rings, barely visible, appeared on the vial wall upon complete solvent evaporation. The vial was then filled with diethyl ether, and the vial walls were vigorously scratched with a metal spatula to crack the initially oily product, yielding a white powder (yield = 80 %). The powder was stored in diethyl ether prior to further use.

Stability test of trimeric Sc^{3+} cluster in acetic acid and $Sc(OTf)_3$. Dried powder of the Sc^{3+} cluster (4 mg, 3.6 μmol) was dissolved in DMF-d_7 (0.55 mL). 6 M acetic acid (100 μL) or 8.5 mM $Sc(OTf)_3$ solution in DMF-d_7 (50 μL, 0.42 μmol; 0.02 equiv. to aldehyde group) was added to the Sc^{3+} cluster solution. Each solution was analyzed using ^{1}H NMR.

S2.3 Extension of trimeric Sc^{3+} cluster

Reaction of trimeric Sc^{3+} cluster with aniline. Dried powder of the Sc^{3+} cluster (2.0 mg, 1.8 μmol) was dissolved in DMF-d_7 (0.5 mL). Aniline (1 μL, 11 μmol; 1 equiv. to aldehyde group) and 8.5 mM $Sc(OTf)_3$ solution in DMF-d_7 (25 μL, 0.21 μmol; 0.02 equiv. to aldehyde group) were added to the Sc^{3+} cluster solution. After mixing well, the solution was moved to an NMR tube and heated in an oven at 70 °C for 3 days. ^{1}H NMR spectra were collected after 1, 2, and 3 days of heating.

Synthesis of BCN-40. The Sc^{3+} cluster was gently dried in air after decanting diethyl ether. (Note: The Sc^{3+} cluster changed to insoluble solid when exposed to air for long time or when soaked and dried repeatedly.) To a 5 mL vial, the Sc^{3+} cluster (2.0 mg, 1.8 μmol), 1,4-dioxane (0.2 mL) and aniline (9 μL, 98 μmol; 9 equiv. to aldehyde group) were added. Separately, an 85 mM $Sc(OTf)_3$ solution was prepared by dissolving $Sc(OTf)_3$ (21.0 mg) in 1,4-dioxane (0.5 mL). Then, the $Sc(OTf)_3$ solution (25 μL, 2.1 μmol; 0.2 equiv. to aldehyde group) was added into the mixture with the Sc^{3+} cluster. After sonication, a pale-yellow solution was obtained. In another vial, TAPP (1.8 mg, 2.7 μmol) was sonicated first with DMF (50 μL), and then with additional 1,4-dioxane (0.15 mL). The TAPP solution (0.2 mL) was added into the solution with the Sc^{3+} cluster. After thorough mixing, the vial was sealed with Teflon tape and heated in an oven at 85 °C for 3 days. After cooling to room temperature, the obtained dark reddish-purple solid was washed and stored in acetone.

S2.4 Vitamin B_{12} adsorption in BCN-40

A BCN-40 suspension (~3.2 mg in 0.2 mL of MeOH) was added into a 1.0 mL of vitamin B_{12} solution in MeOH (3 mg/mL, 2.21 mM). UV-vis spectra were taken at different time intervals (0,

0.1, 0.2, 0.5, 1.5, 4, and 16 hours) to determine the concentration of vitamin B_{12} (λ = 361 nm, ε = 26500 L mol^{-1} cm^{-1}).

Section S3. Supplementary Figures and Tables

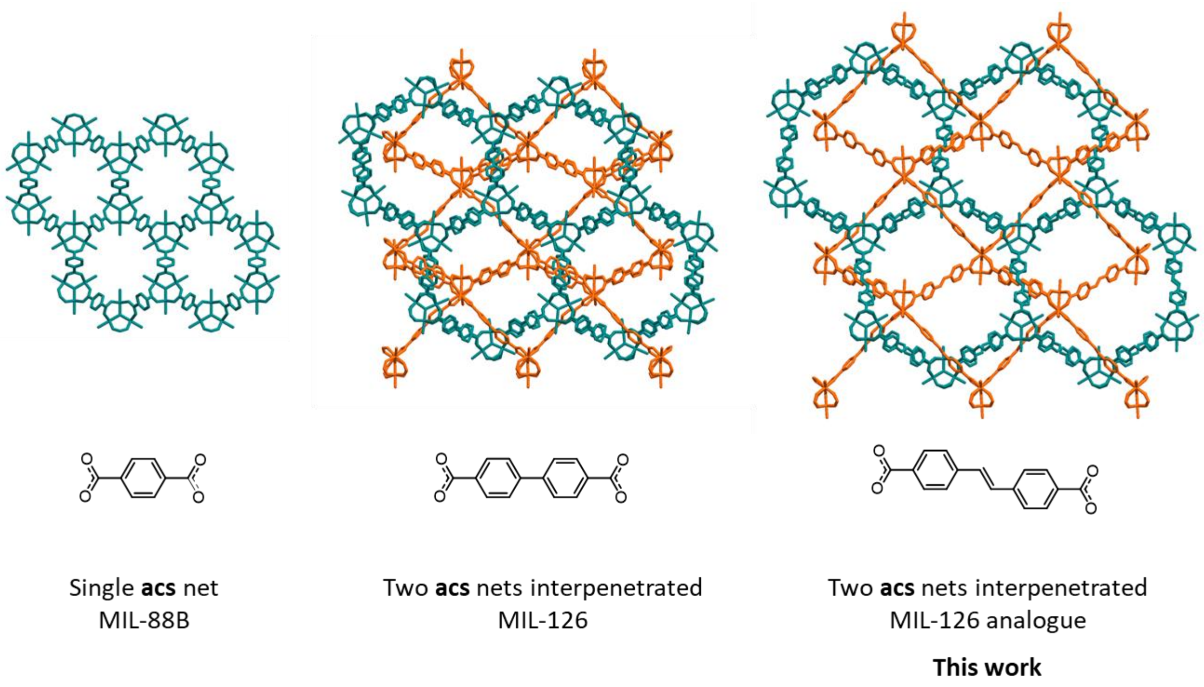

Figure S1. Structures of MIL-88B[3] (CCDC 1415803), MIL-126[4] (CCDC 791671), and MIL-126 analog (model structure) synthesized in this work.

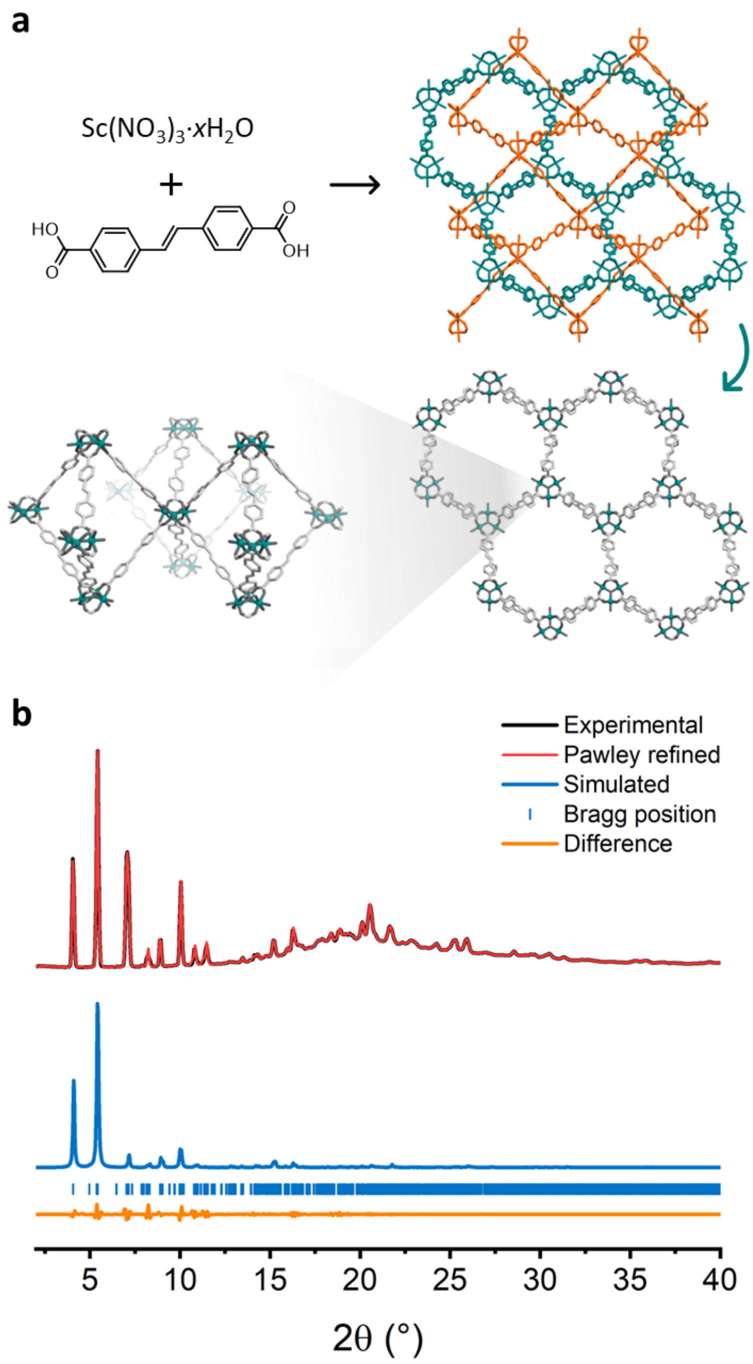

Figure S2. (a) Proposed structure of MIL-126 analog synthesized with $Sc(NO_3)_3 \cdot xH_2O$ and H_2Sti. The model structure was built based on the reported structure of MIL-126 with $P4_32_12$ space group.[4] (b) Experimental PXRD pattern of MIL-126 analog collected using synchrotron radiation (λ = 0.82652 Å) and the refined Pawley fitting (R_p = 1.4%, R_{wp} = 3.5%, a = 24.993(2) Å, c = 42.414(6)).

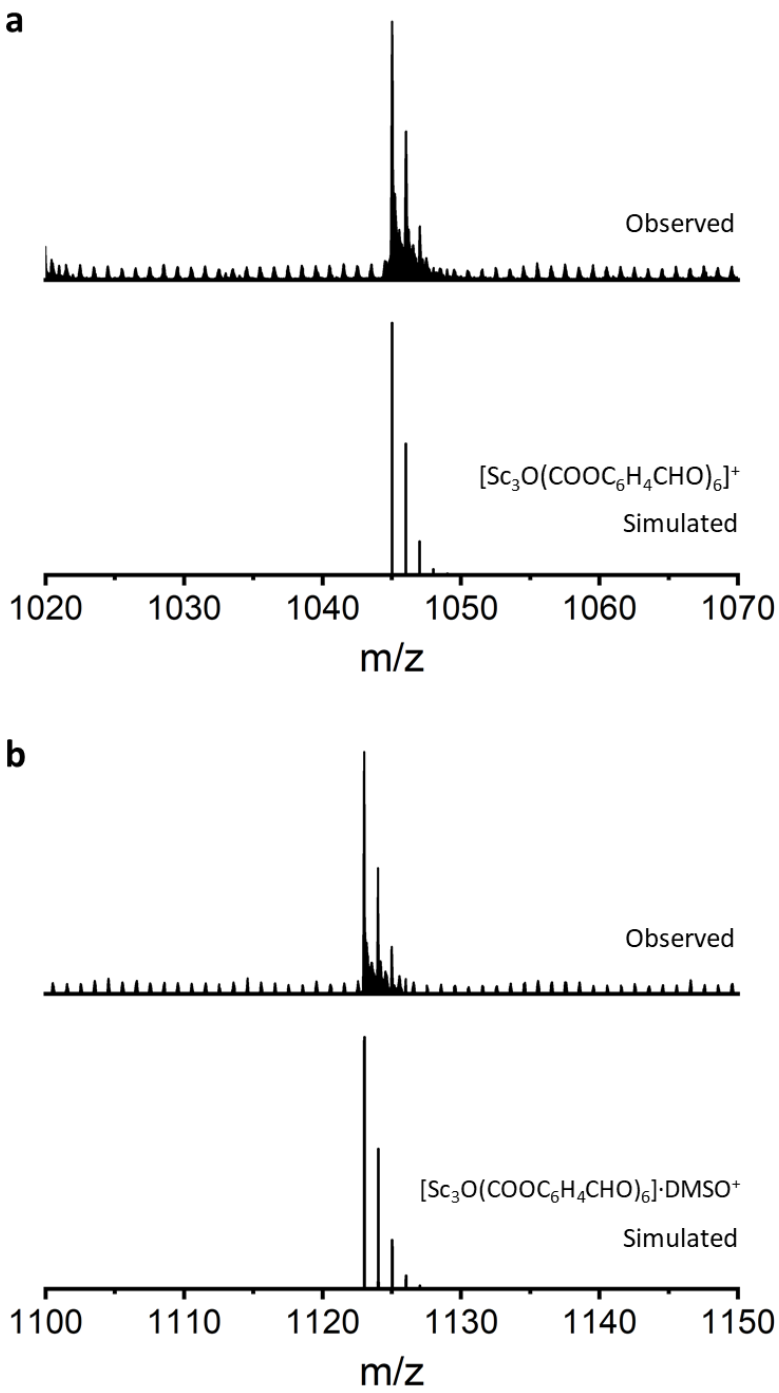

Figure S3. MALDI-MS data for trimeric Sc^{3+} cluster, compared with the simulated isotope pattern of (a) the expected Sc^{3+} cluster and (b) the Sc^{3+} cluster with one additional DMSO molecule.

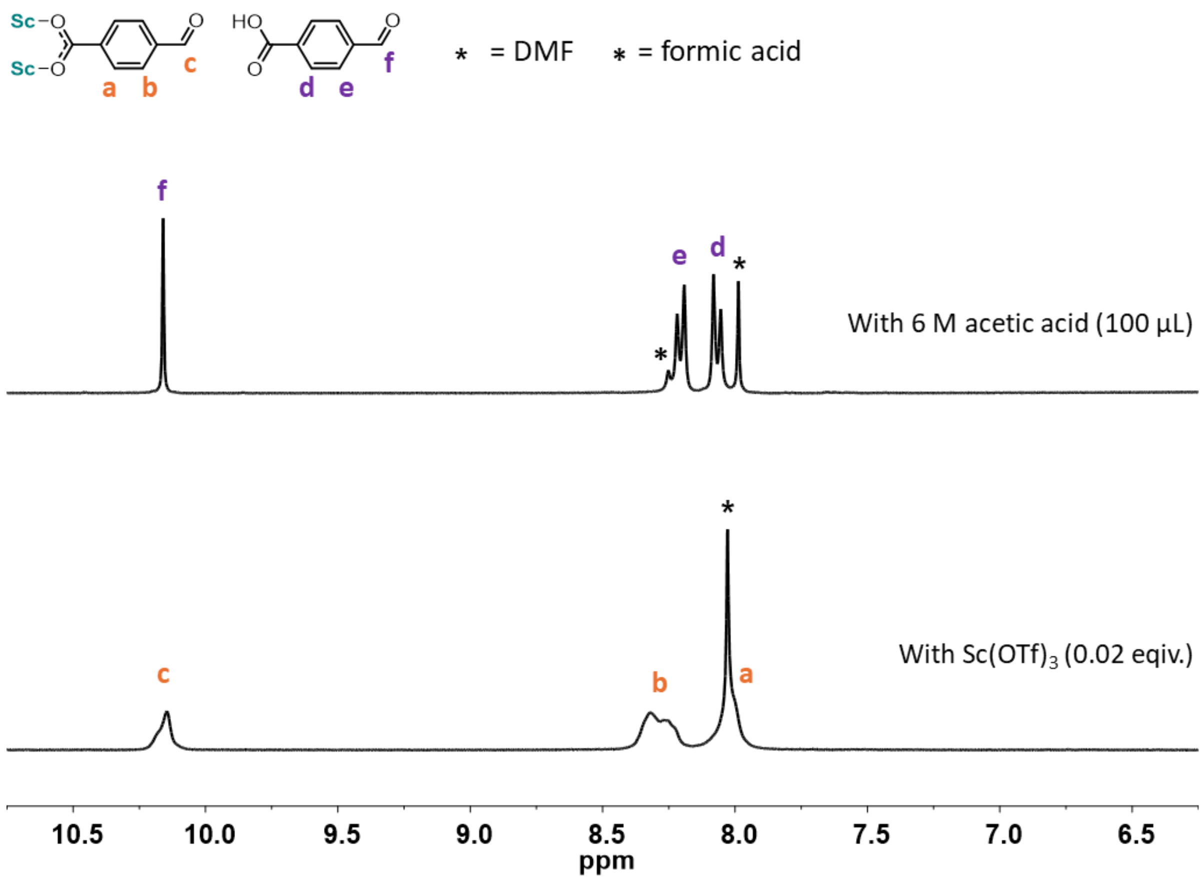

Figure S4. ^{1}H NMR spectra of trimeric Sc^{3+} cluster solution in DMF-d_7 with $Sc(OTf)_3$ (bottom) and 6 M acetic acid (top). The Sc^{3+} cluster with $Sc(OTf)_3$ showed broad peaks, suggesting the integrity of the cluster. In contrast, sharp peaks of 4-formylbenzoic acid were observed with 6 M acetic acid, suggesting the decomposition of the Sc^{3+} cluster. Asterisks indicate formic acid and DMF. Formic acid is produced by hydrolysis of DMF during the MOF synthesis.

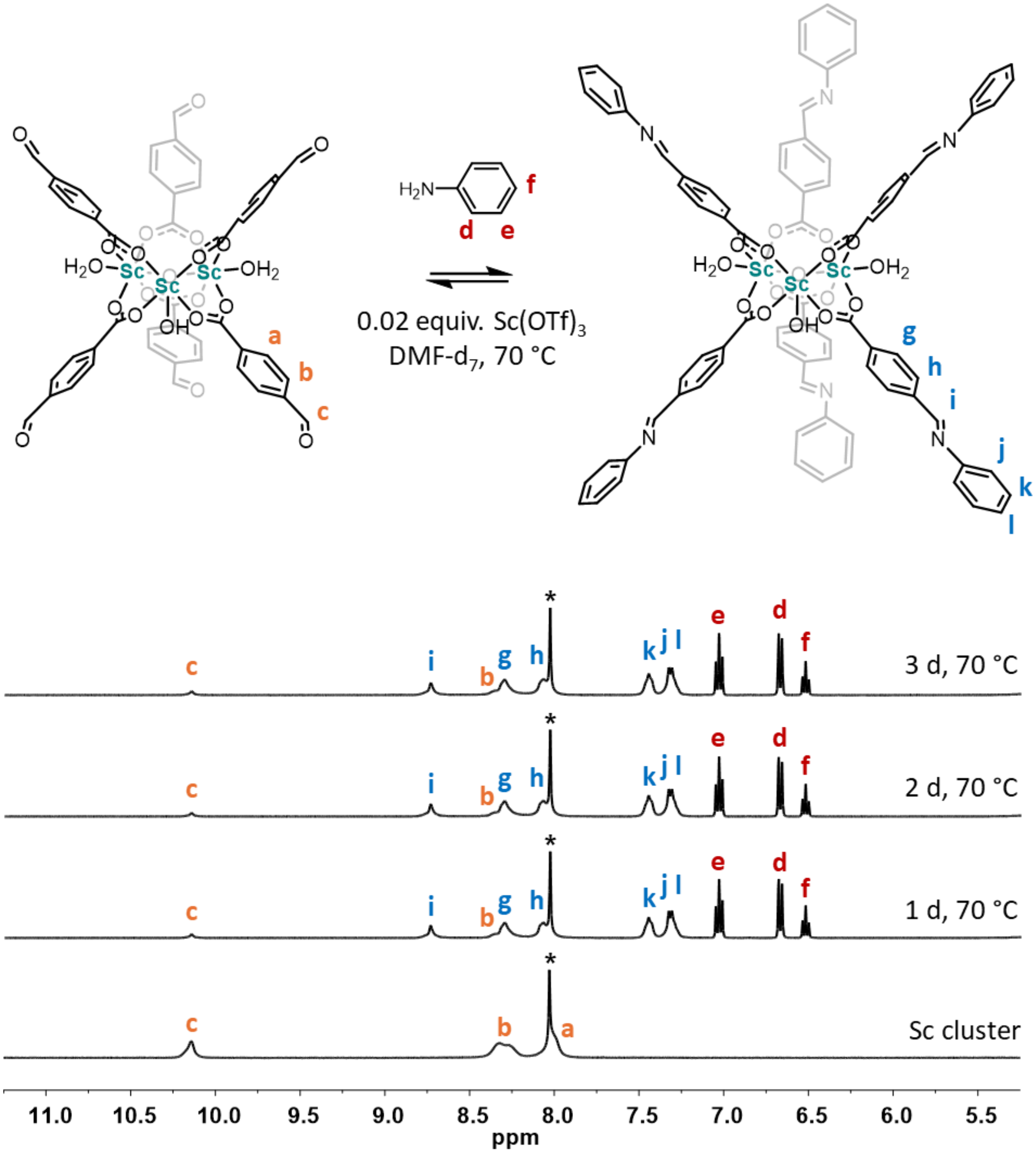

Figure S5. ^{1}H NMR spectra of a reaction mixture with trimeric Sc^{3+} cluster, aniline, $Sc(OTf)_3$, and DMF-d_7. The spectra were collected every 24 hours during the heating at 70 °C for 3 days. 80% conversion from aldehyde into imine groups was observed after 1 day and retained over the next two days. Asterisks indicate DMF.

Table S1. Observed species in MALDI MS data of trimeric Sc^{3+} cluster reacted with aniline.

Chemical formula	The number of reacted aniline per Sc cluster	Theoretical *m*/*z*	Observed *m*/*z*
$Sc_3O(COOC_6H_4CHNC_6H_5)_6$	6	1495.3	1495.5
$Sc_3O(COOC_6H_4CHNC_6H_5)_5(COOC_6H_4CHO)$	5	1420.2	1420.4
$Sc_3O(COOC_6H_4CHNC_6H_5)_4(COOC_6H_4CHO)_2$	4	1345.2	1345.3
$Sc_3O(COOC_6H_4CHNC_6H_5)_3(COOC_6H_4CHO)_3$	3	1270.1	1270.3
$Sc_3O(COOC_6H_4CHNC_6H_5)_2(COOC_6H_4CHO)_4$	2	1195.1	1195.4

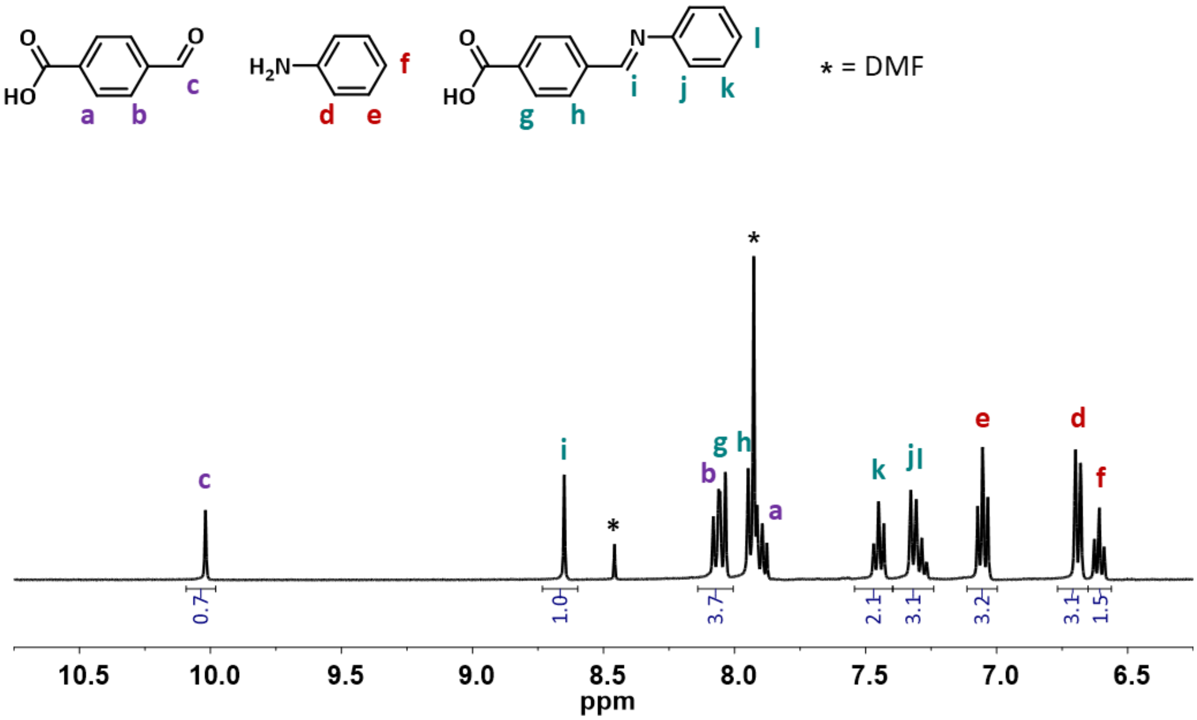

Figure S6. ^{1}H NMR spectrum of the reaction solution (Sc^{3+} cluster, aniline, $Sc(OTf)_3$, and DMF-d_7, 70 °C, 3 days) after digestion with cesium fluoride solution in D_2O (24 mg of CsF in 0.25 mL of D_2O). Compared to a spectrum Figure S5, less ratio of imine bond is observed due to the hydrolysis of this bond by D_2O.

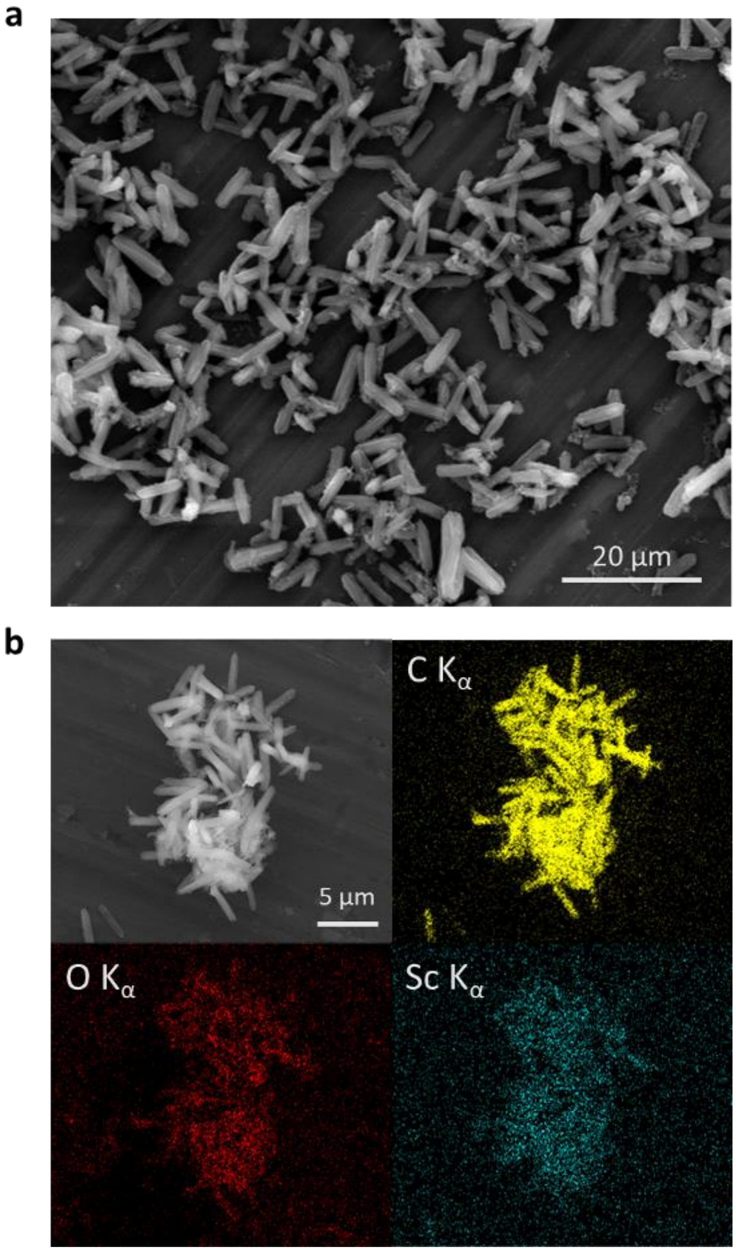

Figure S7. (a) SEM image of BCN-40. (b) EDX elemental mapping of BCN-40 showing the C (yellow), O (red), and Sc (cyan) distribution.

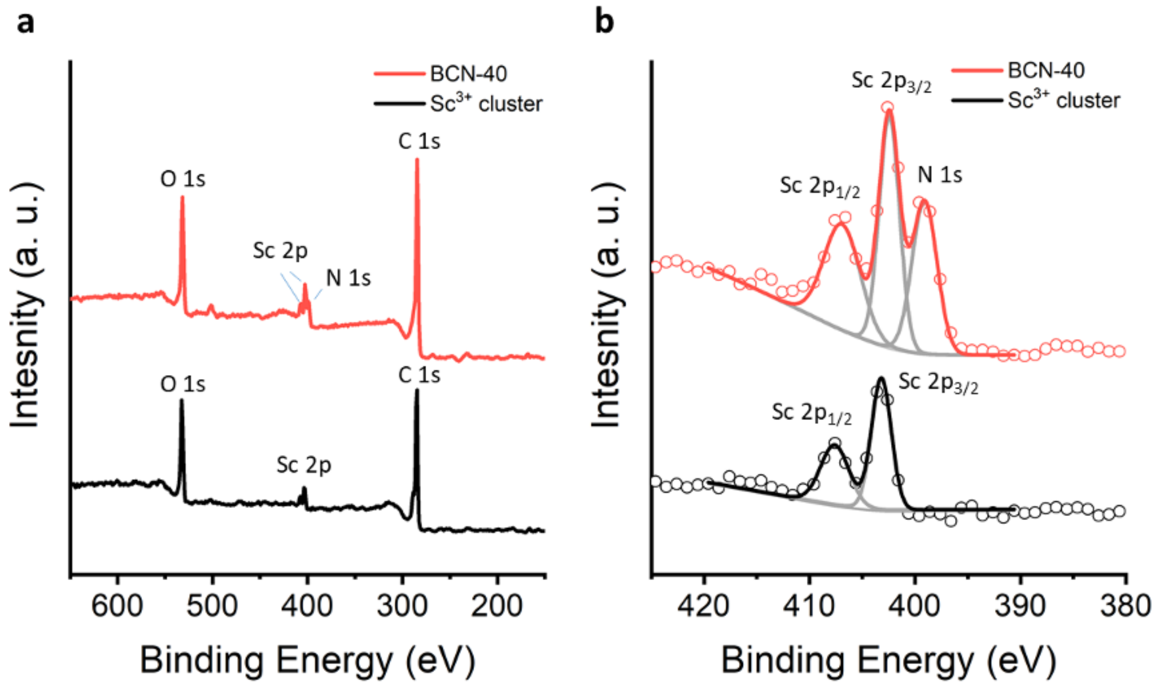

Figure S8. (a) XPS spectra of Sc^{3+} cluster and BCN-40. Sc(III) oxidation state and coordination environment are maintained during the reaction. (b) Magnified XPS spectra for Sc 2p and N 1s peak analysis. The N 1s peak at 399 eV confirms the presence of TAPP in BCN-40.

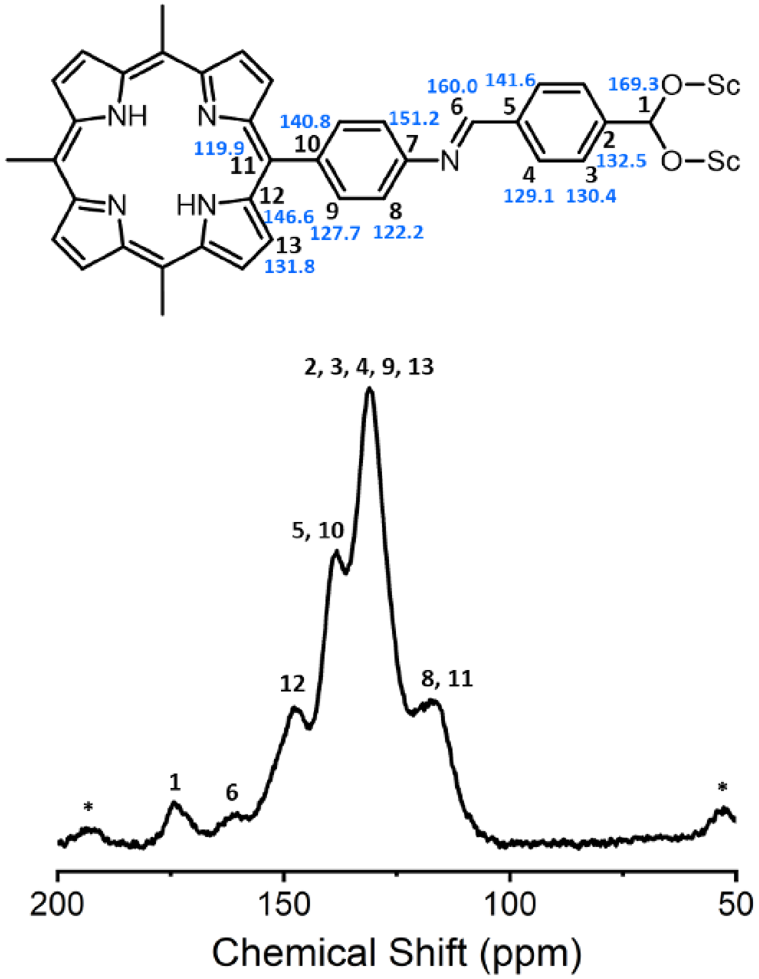

Figure S9. ^{13}C CP-MAS solid-state NMR of BCN-40. Asterisks (*) indicate peaks arising from spinning side bands. The imine peak was observed at 161.0 ppm, in agreement with that observed in analogous covalent organic frameworks (COFs) built from TAPP linker.[5]

Table S2. Hexagonal channel sizes in examples of MOFs with **stp** topology.

Name	Hexagonal channel size[a]	Reference
BCN-40	4.7 nm	This work
PCN-600	3.1 nm	6
MOF-892	3.0 nm	7
NiTCPE-*stp*	2.6 nm	8
USTC-9	2.2 nm	9

[a]Distance between two organic ligands facing each other in the hexagonal channel.

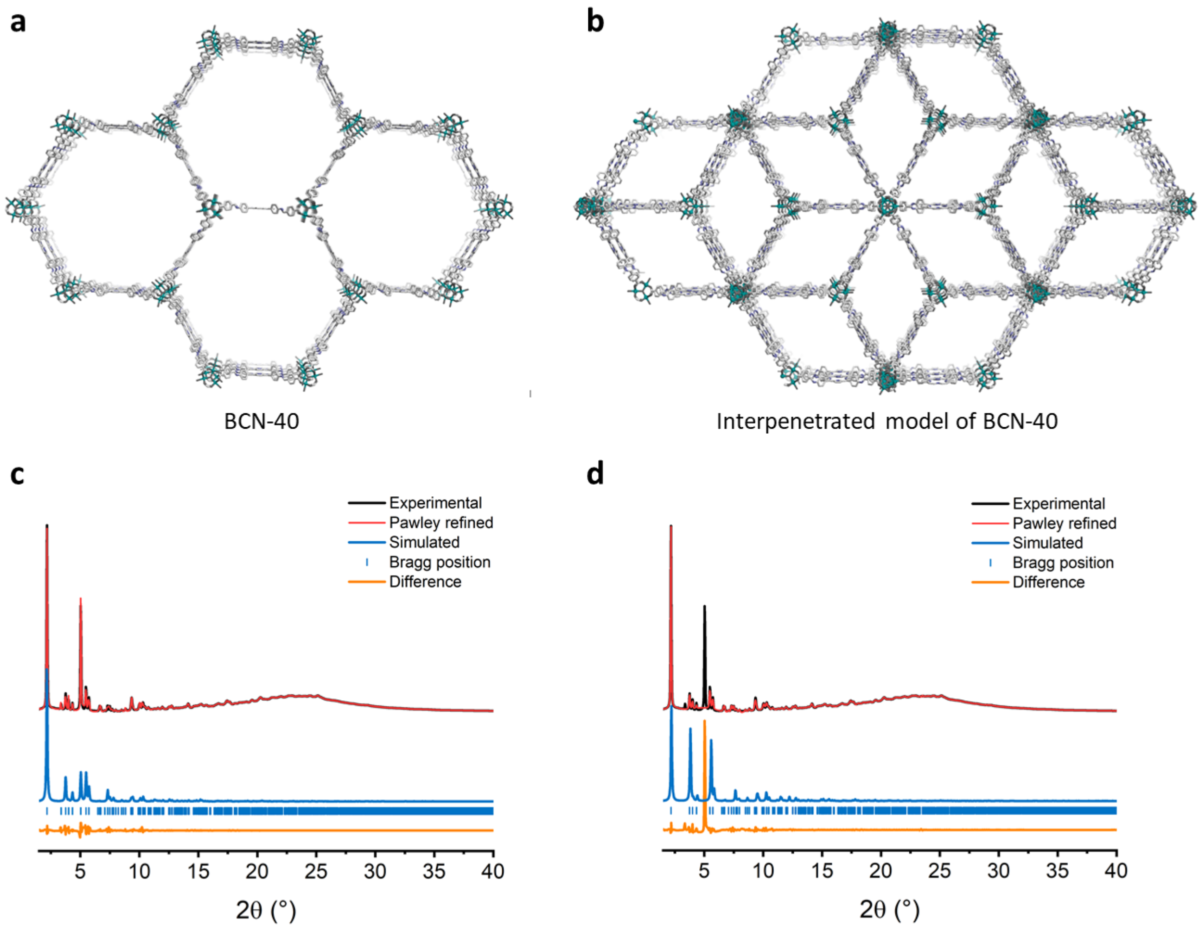

Figure S10. Comparison of experimental PXRD pattern of BCN-40 with simulated patterns of non-interpenetrated and interpenetrated models. (a) Structure of non-interpenetrated BCN-40 modeled in *P*6/*m* space group. (b) Structure of interpenetrated BCN-40 modeled in *P*-31*c* space group. (c) Pawley fitting of experimental PXRD pattern with the non-interpenetrated model (R_p = 1.6%, R_{wp} = 4.4%). (d) Pawley fitting of experimental PXRD pattern with the interpenetrated model (R_p = 3.4%, R_{wp} = 12.8%). A significant discrepancy between the experimental and fitted patterns was observed at 2θ = ~5°.

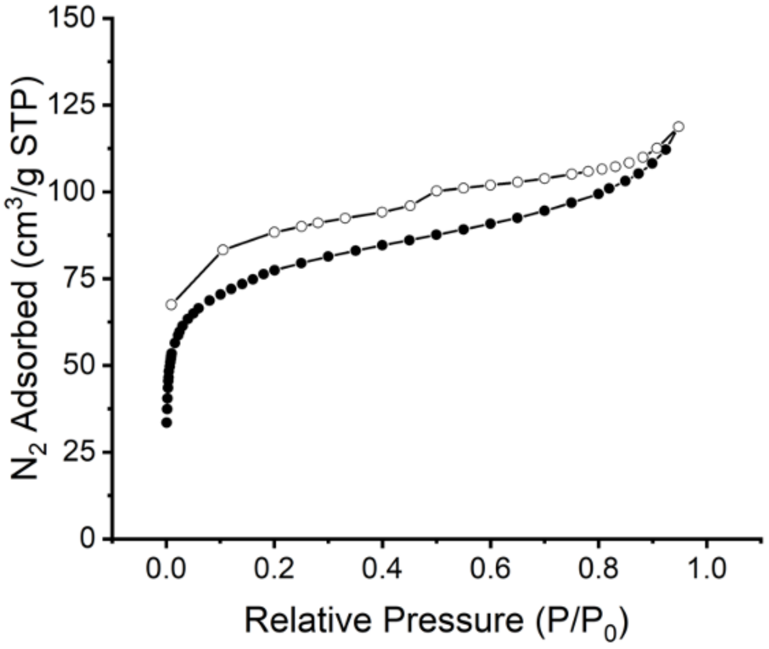

Figure S11. N_2 adsorption isotherm of BCN-40 at 77 K.

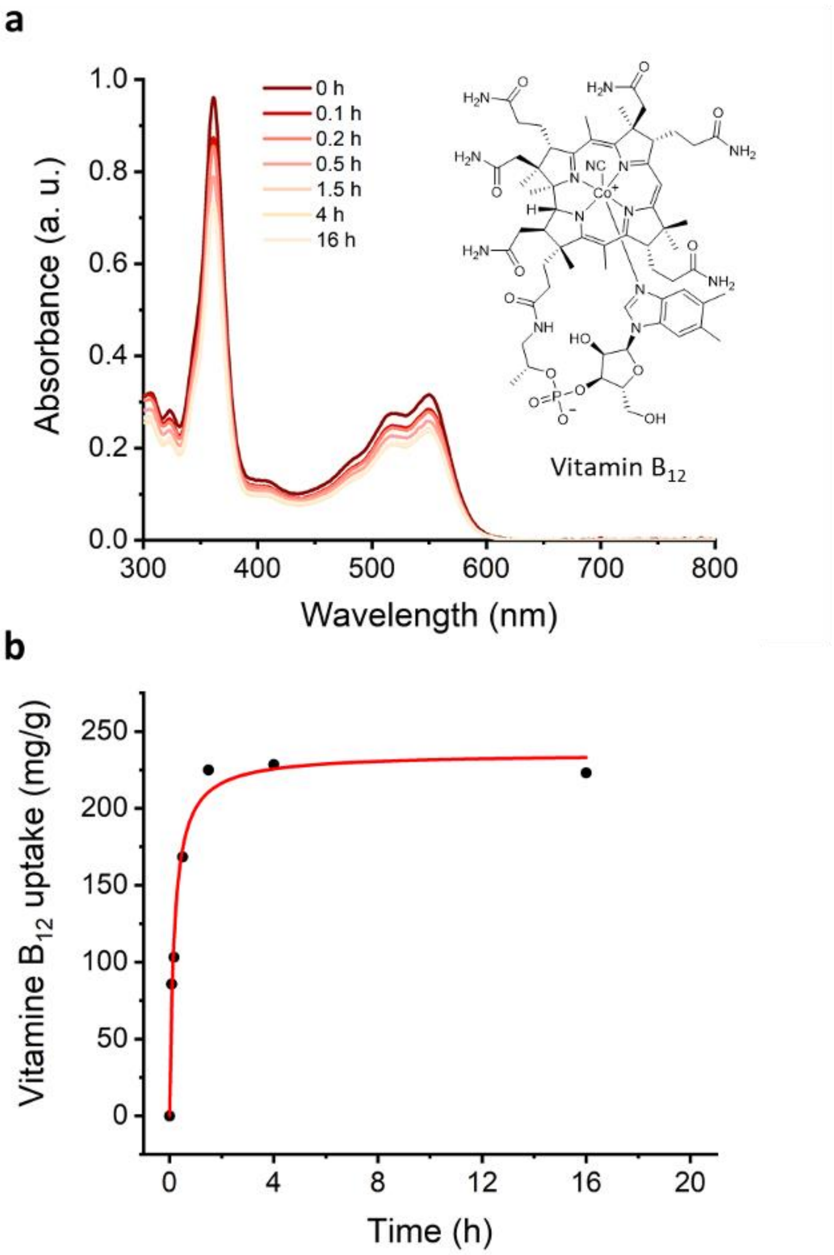

Figure S12. (a) UV-vis spectra for vitamin B_{12} adsorption into BCN-40 at different times. (b) Adsorption profile of vitamin B_{12} into BCN-40, fitted with the pseudo-second-order kinetic model.

References

1. Juanhuix, J.; Gil-Ortiz, F.; Cuní, G.; Colldelram, C.; Nicolás, J.; Lidón, J.; Boter, E.; Ruget, C.; Ferrer, S.; Benach, J. Developments in Optics and Performance at BL13-XALOC, the Macromolecular Crystallography Beamline at the Alba Synchrotron. *J. Synchrotron Radiat.* **2014**, *21* (4), 679–689.
2. A P Hammersley, *ESRF Internal Report*, **ESRF97HA02T**, "FIT2D: An Introduction and Overview", (1997).
3. Wei, Y.-S.; Zhang, M.; Liao, P.-Q.; Lin, R.-B.; Li, T.-Y.; Shao, G.; Zhang, J.-P.; Chen, X.-M. Coordination Templated [2+2+2] Cyclotrimerization in a Porous Coordination Framework. *Nat. Commun.* **2015**, *6* (1), 8348.
4. Dan-Hardi, M.; Chevreau, H.; Devic, T.; Horcajada, P.; Maurin, G.; Férey, G.; Popov, D.; Riekel, C.; Wuttke, S.; Lavalley, J.-C.; Vimont, A.; Boudewijns, T.; de Vos, D.; Serre, C. How Interpenetration Ensures Rigidity and Permanent Porosity in a Highly Flexible Hybrid Solid. *Chem. Mater.* **2012**, *24* (13), 2486–2492.
5. Gong, C.; Yang, X.; Wei, X.; Dai, F.; Zhang, T.; Wang, D.; Li, M.; Jia, J.; She, Y.; Xu, G.; Peng, Y. Three-Dimensional Porphyrin-Based Covalent Organic Frameworks with stp Topology for an Efficient Electrocatalytic Oxygen Evolution Reaction. *Mater. Chem. Front.* **2023**, *7* (2), 230–237.
6. Wang, K.; Feng, D.; Liu, T.-F.; Su, J.; Yuan, S.; Chen, Y.-P.; Bosch, M.; Zou, X.; Zhou, H.-C. A Series of Highly Stable Mesoporous Metalloporphyrin Fe-MOFs. *J. Am. Chem. Soc.* **2014**, *136* (40), 13983–13986.
7. Nguyen, P. T. K.; Nguyen, H. T. D.; Nguyen, H. N.; Trickett, C. A.; Ton, Q. T.; Gutiérrez-Puebla, E.; Monge, M. A.; Cordova, K. E.; Gándara, F. New Metal–Organic Frameworks for Chemical Fixation of CO_2. *ACS Appl. Mater. Interfaces* **2018**, *10* (1), 733–744.
8. Zheng, H.-L.; Zhao, J.-Q.; Sun, Y.-Y.; Zhang, A.-A.; Cheng, Y.-J.; He, L.; Bu, X.; Zhang, J.; Lin, Q. Multilevel-Regulated Metal–Organic Framework Platform Integrating Pore Space Partition and Open-Metal Sites for Enhanced CO_2 Photoreduction to CO with Nearly 100% Selectivity. *J. Am. Chem. Soc.* **2023**, *145* (50), 27728–27739.
9. Han, W.; Ma, X.; Wang, J.; Leng, F.; Xie, C.; Jiang, H.-L. Endowing Porphyrinic Metal–Organic Frameworks with High Stability by a Linker Desymmetrization Strategy. *J. Am. Chem. Soc.* **2023**, *145* (17), 9665–9671.
